# Impact of Electronic Chronic Pain Questions on patient-reported outcomes and healthcare utilization, and attitudes toward eCPQ use among patients and physicians: prospective pragmatic study in a US general practice setting

**DOI:** 10.3389/fmed.2023.933975

**Published:** 2023-06-22

**Authors:** Lois Lamerato, Vinay Shah, Lucy Abraham, Joseph C. Cappelleri, Bonnie DeLor, Stacy R. Ellsworth, Rozelle Hegeman-Dingle, Peter W. Park

**Affiliations:** ^1^Henry Ford Health, Detroit, MI, United States; ^2^Pfizer Ltd., Tadworth, Surrey, United Kingdom; ^3^Pfizer Inc., Newington, CT, United States; ^4^Pfizer Inc., New York, NY, United States

**Keywords:** chronic pain, Electronic Chronic Pain Questions, eCPQ, patient-reported outcomes, healthcare resource utilization, primary care setting

## Abstract

**Objective:**

The Electronic Chronic Pain Questions (eCPQ) has been developed to help healthcare providers systematically capture chronic pain data. This study evaluated the impact of using the eCPQ on patient-reported outcomes (PROs) and healthcare resource utilization (HCRU) in a primary care setting, and patient and physician perceptions regarding use of, and satisfaction with, the eCPQ.

**Methods:**

This was a prospective pragmatic study conducted at the Internal Medicine clinic within the Henry Ford Health (HFH) Detroit campus between June 2017 and April 2020. Patients (aged ≥18 years) attending the clinic for chronic pain were allocated to an Intervention Group to complete the eCPQ in addition to regular care, or a control group to receive regular care only. The Patient Health Questionnaire-2 and a Patient Global Assessment were assessed at baseline, 6-months, and 12-months study visits. HCRU data were extracted from the HFH database. Telephone qualitative interviews were conducted with randomly selected patients and physicians who used the eCPQ.

**Results:**

Two hundred patients were enrolled, 79 in each treatment group completed all 3 study visits. No significant differences (*p* > 0.05) were found in PROs and HCRU between the 2 groups. In qualitative interviews, physicians and patients reported the eCPQ as useful, and using the eCPQ improved patient-clinician interactions.

**Conclusion:**

Adding the eCPQ to regular care for patients with chronic pain did not significantly impact the PROs assessed in this study. However, qualitative interviews suggested that the eCPQ was a well-accepted and potentially useful tool from a patient and physician perspective. By using the eCPQ, patients were better prepared when they attended a primary care visit for their chronic pain and the quality of patient-physician communication was increased.

## 1. Introduction

Chronic pain is a worldwide health problem (1–3). In the US, chronic pain was reported in 20.4% of adults in 2019, with 7.4% of adults reporting limited life or work activities caused by chronic pain (1); 12% of adults receiving care in a large, integrated healthcare system had chronic pain (4). For individuals, the presence of chronic pain is associated with substantial disability, poor mental health, lower quality of life, and decreased work function (5, 6). Besides the adverse effects of chronic pain on the patient’s life, the social and family environment of the patient has also been negatively affected (7). For society, chronic pain constitutes a significant burden on the healthcare system, resulting in substantial utilization of resources and costs (8–12).

Pain is a sensory and emotional experience; biological, psychological, and social factors can influence pain reporting (13). Based on pain pathophysiology, nociceptive, neuropathic, and sensory hypersensitivity or fibromyalgia-like pain are the 3 main types of pain (14, 15). Various patient-reported outcome (PRO) questionnaires have been used to assess pain by traditional (paper-and-pencil, telephone, or in-person) or electronic data capture methods (16). Different PROs have been recommended for different patient populations (17–19). The influential factors of pain perception and the impact of patient psychological characteristics on chronic pain management have been acknowledged (20–22). Considering the complex nature of pain, a comprehensive pain assessment including pain typing will enable effective treatment and monitoring strategies.

The Electronic Chronic Pain Questions (eCPQ)^1^ module has been developed to help healthcare providers systematically capture chronic pain data in their electronic health records (EHR), guiding their “real time” clinical evaluation and allowing them to track patients’ pain progress over time. The eCPQ was developed based on guidelines on chronic pain management through literature review and consultation with patients and clinicians. The eCPQ module has 14 items (Table 1; Supplementary Figure S1) to determine the presence of chronic pain (defined as pain that persists for ≥3 months, beyond the normal time of healing) and assesses pain intensity, location, and pain-related interference with function, sleep, and mood. The ID Pain^®^ Screener and the sensory hypersensitivity pain questions are part of the eCPQ to assist the provider in determining pain quality and type. A cross-sectional study in adults with a physician-confirmed diagnosis of either a neuropathic pain or sensory hypersensitivity condition found that a paper version of the CPQ was able to help differentiate between patients with neuropathic pain and those with sensory hypersensitivity (23), and psychometric evaluation of the eCPQ demonstrated it was valid and reliable for use in the primary care setting (24).

**Table 1:** Questionnaires used in this study.

Name	Purpose	Evaluation period	Content	Output
eCPQ	Assessing patient’s experience regarding chronic pain	Within 1 week prior to evaluation for all questions except for Question 1, which has a 3-month reference period	14 items on 6 aspects (1) Does the patient have chronic pain (defined as pain that persists for ≥3 months, beyond the normal time of healing)? (2) What is the pain intensity? (0–10 on NRS) (3) Where is the pain located? (4) Does the pain interfere with usual daily activities, sleep, and mood? (0–10 on NRS) (5) What is the quality and the type of the pain? Is it nociceptive or neuropathic? (6) Is there sensory hypersensitivity according to the assessment performed in combination with clinical examination and patient medical history?	Scores of pain intensity and interference of pain: ranging from 0 to 10 Pain category by the intensity score: Mild: 0–3 Moderate: 4–6 Severe: 7–10 Type of pain: assessed by items 5 to 9 Nociceptive pain: “yes” for <3 items Neuropathic pain: “yes” for ≥3 items (if no response provided for any of the items, “no” was assumed as the choice)
PGA	Self-rating of overall health condition considering everything affected by the pain	On the day of evaluation	1 item (5-point Likert scale) Conditions from “very good” (asymptomatic and no limitation of normal activities), “good” (mild symptoms and no limitation of normal activities), “fair” (moderate symptoms and limitation of some normal activities), “poor” (severe symptoms and inability to carry out most normal activities), to “very poor” (very severe symptoms which are intolerable and inability to carry out all normal activities)	Score: ranging from 1 to 5 Very good: 1 Good: 2 Fair: 3 Poor: 4 Very poor: 5
PHQ-2	Aiming to detect the frequency of the depressed mood and anhedonia	Within 2 weeks prior to evaluation	First 2 items of the PHQ-9 (0–3 for each item) Frequency from “not at all,” “several days,” “more than half the days,” to “nearly every day”	Score: ranging from 0 to 6 A score ≥3 is considered as positive and warrants further assessment with PHQ-9
PHQ-9	Screening, diagnosing, monitoring, and measuring the severity of depression	Within 2 weeks prior to evaluation	9 items (0–3 for each item) Frequency from “not at all,” “several days,” “more than half the days,” to “nearly every day”	Score: ranging from 0 to 27 Depression category by PHQ-9 score: Minimal symptom: 5–9 Minor depression: 10–14 Major depression moderately severe: 15–19 Major depression: >20

eCPQ, electronic Chronic Pain Questions; NRS, numeric rating scale; PGA, Patient Global Assessment; PHQ-2, Patient Health Questionnaire-2; PHQ-9, Patient Health Questionnaire-9.

Consistent, systematic electronic capture of data for chronic pain, such as the Collaborative Health Outcomes Information Registry (CHOIR), allows physicians to assess and monitor patients more easily with improved guidance on treatment decisions. These data will also allow healthcare organizations to more accurately estimate the prevalence impact of chronic pain on healthcare resource utilization (HCRU) trends. Meanwhile, systematically obtaining pain-related data could enhance the interaction between the patient and the clinician, leading to a good patient-clinician relationship that can result in effective pain management. The current study aimed to determine if using the eCPQ in a primary care practice setting within a large integrated delivery network (the Henry Ford Health; HFH) can lead to improved PROs. The impact of the implementation of the eCPQ on HCRU was assessed. The patients’ and the physicians’ perceptions about the use, feasibility, and satisfaction with the eCPQ were evaluated.

## 2. Materials and methods

This was a prospective pragmatic study conducted at the Internal Medicine clinic within the HFH Detroit campus. The study protocol was approved by the Institutional Review Board at HFH. The study was conducted in compliance with the Declaration of Helsinki. All participating patients provided written informed consent.

### 2.1. Patient population

Eligible patients were men and women aged ≥18 years who were attending a primary care visit for their chronic pain. A diagnosis of “chronic pain” (non-cancer) made by physicians in the patient Problem List was required (see Supplementary Table S1 for the eligible International Classification of Diseases, 10th edition, Clinical Modification codes; to capture a broad range of chronic pain conditions, all patients with a generic, high-level diagnosis of “chronic pain” were eligible). Willingness to participate in a 1-year study and ability to speak, read, and write English sufficiently well to complete study questionnaires were required. For the Intervention Group, eligible patients were required to respond “yes” to Question 1 of the eCPQ at the baseline visit to confirm that they had been experiencing pain on most days or every day during the past 3 months and be willing and able to participate in a one-time telephone interview.

Patients who were not willing or able to participate in an interview were not eligible.

### 2.2. Study procedures

**Figure 1:**
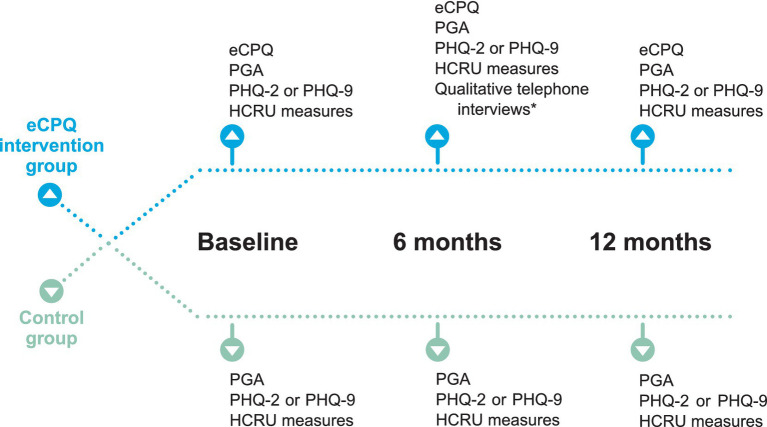
Schematic of assessments performed at each study visit. *Approximately 20% of participants were randomly assigned to the qualitative interview using a 1:5 randomized block design. Randomization was performed at the time of assignment of unique patient ID. Efforts were made to schedule the qualitative interview within 2 weeks of Visit 2. eCPQ, Electronic Chronic Pain Questions; HCRU, healthcare resource utilization; PGA, Patient Global Assessment; PHQ, Patient Health Questionnaire.

Patients scheduled for their regular pain visit were approached by clinical research personnel via telephone prior to their visit to determine their interest and eligibility for the study. Eligible patients going to the North area of the clinic for their visits were in the Intervention Group, whereas those going to the South area of the clinic were in the Control Group. Patients in the Intervention Group completed the eCPQ in addition to regular care and patients in the control group received regular care only (Figure 1). There were no differences in clinic procedures; patient flow; and attending physician expertise, specialty, or experience between the 2 clinic areas. Attending physicians seeing patients for 1 group did not have contact with patients in the other group.

Patients were to be seen in the clinic at least once every 6 months for their chronic pain as per standard of care. There were 3 planned study visits, Visit 1 at baseline, Visit 2 at 6 ± 2 months, and Visit 3 at 12 months. All visits were dependent on patients’ needs (or actions) and availability of appointments. Patients in the Intervention Group self-completed a paper version of the eCPQ in the waiting room before seeing the physician at each visit. The physician seeing the patient reviewed the eCPQ answers with the patient during their appointment, and the clinical research personnel input the data into the electronic medical records. At Visits 2 and 3, patients in the Intervention Group were asked to complete all the questions of the eCPQ regardless of their answers to Question 1.

The Patient Health Questionnaire-2 (PHQ-2) was completed routinely as part of the standard of care in the usual (non-study) visits to screen for depression. For those who had PHQ-2 scores indicative of a need for further assessment, the PHQ-9 was also completed. As part of the regular care, patients in both groups completed the Patient Global Assessment (PGA) following their appointment at each visit (Table 1).

To capture the clinician and patient perspectives of the eCPQ, approximately 15 patients in the Intervention Group were randomly assigned to a qualitative interview using a 1:5 randomized block design; approximately 10 clinicians were randomly selected among those who used the eCPQ and interviewed as well. The patient interview was scheduled after Visit 2 to obtain any insights regarding the use of eCPQ from the patient-perspective in a timely manner. The physicians’ interviews were scheduled after the last study patient was enrolled and approximately 50% of study participants had completed Visit 2.

### 2.3. eCPQ-specific assessments

Changes in eCPQ pain intensity and pain interference with mood, sleep, and usual activities from baseline to 6 and 12 months were assessed. Percentage of the patients who had neuropathic, nociceptive, or sensory hypersensitivity type of pain (based solely on patient responses to the eCPQ) at baseline, 6 months, and 12 months were summarized.

The qualitative interviews were conducted by telephone by a trained independent interviewer (Evidera, Bethesda, MD, USA; hired by the study sponsor). Patients and physicians were asked a series of open-ended questions (Supplementary Tables S2, S3) about their perceptions of the eCPQ, how the eCPQ impacted their care, areas of improvement in the process, and perceived value. The patient interview lasted for approximately 60 min and the clinician interview lasted no more than 30 min. All interviews were audio-recorded with the interviewees’ permission, and subsequently transcribed. Qualitative data from the interviews were reviewed by the interviewer, and key themes that described important concepts raised by participants were identified. An *a priori* coding dictionary was developed based on themes and concepts that emerged during the interviews. ATLAS.ti software version 8.4.20.0. was used to facilitate the systematic coding and analysis of the data. The findings of the interviews were summarized.

### 2.4. Outcomes

Changes in PGA score and PHQ-2 and PHQ-9 scores from baseline to 6 and 12 months were evaluated for the Intervention vs. the Control Group.

Administrative data regarding all-cause HCRU were downloaded from the electronic data warehouse of HFH. Assessment of HCRU included inpatient admissions, inpatient days, emergency department visits, total outpatient visits, outpatient visit–primary care, outpatient visit–specialty care, outpatient visit–pain management, and total billing charges. HCRU specific to comorbidities was not assessed separately but included in all-cause HCRU. The assessments for HCRU (overall and by pain severity) were performed at 6 and 12 months from the baseline visit.

### 2.5. Statistical methods

No previous research on the primary endpoint of PGA score had been published or documented at the time of the study with respect to the presence versus the absence of the eCPQ intervention. Therefore, a general benchmark on treatment effect size and sample size determination was applied (25). For a treatment effect on PGA score where the difference in mean scores on PGA between the presence and absence of eCPQ was postulated as 0.5 standard deviation (SD), 64 subjects per treatment arm were needed for 80% statistical power (0.05 2-tailed test of significance). Assuming 30% attrition, 92 subjects per arm (64/0.7) were needed to be enrolled.

Descriptive statistics were used. The means, SDs, medians, and sample sizes were reported for continuous outcomes (e.g., PGA, pain, function, mood, sleep, certain types of resource utilization). For categorical outcomes, proportion, counts, and sample sizes were provided. Differences between the 2 groups were assessed by *t*-test or chi-square test. The statistical software used was SPSS version 26. *p* values <0.05 were considered as significantly different.

## 3. Results

### 3.1. Patient population

In total, 200 patients were enrolled between June 2017 and March 2019 (Intervention Group: 103; Control Group: 97). Five patients were excluded from the Intervention Group (consent withdrawal: 3; enrollment error: 2); one patient in the Control Group died before Visit 3 and was excluded (Supplementary Figure S2). Therefore, 98 and 96 patients in the Intervention and the Control Group were analyzed, respectively.

The study was completed in April 2020, 79 patients in both the Intervention (79/98, 80.6%) and the Control Group (79/96, 82.3%) completed all 3 visits.

Baseline demographic characteristics were comparable between the 2 groups, the mean age was over 60 years, most (>60%) patients were female, and over 85% were Black (reflecting the overall population cared for in these urban clinics). There were no significant differences in the percentages of patients using commercial insurance, Medicaid, or Medicare between the 2 groups (Table 2).

**Table 2:** Baseline demographic characteristics.

	Intervention group *n* = 98	Control group *n* = 96	*P* value
Age, years			
<50	12 (12.2)	12 (12.5)	NS
50–59	31 (31.6)	27 (28.1)	
60–69	35 (35.7)	35 (36.5)	
≥70	20 (20.4)	22 (22.9)	
Age, years			
Mean (SD)	60.6 (11.1)	61.6 (10.4)	NS
Median (range)	61.0 (28–89)	61.5 (37–91)	
Gender			
Female	65 (66.3)	61 (63.5)	NS
Male	33 (33.7)	35 (36.5)	
Race/Ethnicity			
White	13 (13.3)	10 (10.4)	NS
Black	85 (86.7)	85 (88.5)	
Hispanic	0 (0.0)	1 (1.0)	
Insurance			
Commercial	15 (15.3)	18 (18.8)	NS
Medicaid	19 (19.4)	24 (25.0)	
Medicare	64 (65.3)	54 (56.3)	

Values are *n* (%) unless stated otherwise. No significant difference was found (*p* > 0.05 for all) between the 2 groups. NS, not significant; SD, standard deviation.

### 3.2. The Electronic Chronic Pain Questions

At baseline, Visit 2, and Visit 3, 97, 77, and 79 patients in the Intervention Group completed the eCPQ form, respectively (Table 3). At each visit, all patients who completed the eCPQ confirmed the presence of chronic pain. Many areas of the body were affected by chronic pain, with the back and the lower extremities as the most frequently reported (Supplementary Table S4). For most items, there were minimal changes from baseline to follow-up visits.

**Table 3:** eCPQ responses.

eCPQ item	Baseline *n* = 97*	Visit 2 *n* = 77	Change from baseline to Visit 2	Visit 3 *n* = 79	Change from baseline to Visit 3
**Confirmation of having chronic pain**					
1. Have you experienced pain on most days in the past 3 months?	Yes – 100%	Yes – 100%	NA	Yes – 100%	NA
**Pain intensity and location**					
2. Select the number that best describes your pain on average over the past week. (0–10), mean (SD)	7.6 (1.9)	7.5 (1.7)	−0.1	7.4 (2.0)	−0.2
Pain severity category, *n* (%)					
Mild (0–3)	2 (2.1)	NE	NA	NE	NA
Moderate (4–6)	23 (23.7)	NE	NA	NE	NA
Severe (7–10)	72 (74.2)	NE	NA	NE	NA
3. Please indicate all areas where you have had pain over the past week	*n* = 95	*n* = 76		*n* = 78	
Mean^†^ number of pain areas (SD)	5.5 (3.9)	5.0 (3.8)	NE	5.3 (4.1)	NE
Range^‡^	0–20	1–23	NE	1–21	NE
**ID PAIN® screener**	Yes responses, *n* (%)^§^				
4. Did the pain feel like pins and needles?	57 (58.8)	49 (64.5)	NA	44 (56.4)	NA
5. Did the pain feel hot/burning?	56 (57.7)	40 (51.9)	NA	39 (50.6)	NA
6. Did the pain feel numb?	54 (55.7)	38 (49.4)	NA	39 (50.0)	NA
7. Did the pain feel like electrical shocks?	44 (45.8)	35 (45.5)	NA	27 (34.6)	NA
8. Is the pain made worse with the touch of clothing or bed sheets?	34 (35.1)	18 (23.4)	NA	21 (27.3)	NA
9. Is the pain limited to your joints?	24 (25.0)	18 (23.4)	NA	9 (11.7)	NA
**Impact of pain on function, sleep, and mood (thinking of the past week)**					
10. How much did pain interfere with your usual activities (such as daily routine, walking, leisure or social activities, work, housework, self-care)? (0–10), mean (SD)	7.3 (2.6)	7.4 (2.0)	+0.2	7.2 (2.58)	−0.04
11. How much did pain interfere with your sleep? (0–10), mean (SD)	7.3 (3.0)	7.5 (2.5)	+0.2	6.7 (3.4)	−0.7
12. How much did pain interfere with your mood? (0–10), mean (SD)	6.4 (3.0)	6.4 (3.1)	−0.1	6.2 (3.0)	−0.2
**Sensory hypersensitivity or fibromyalgia-like pain**					
13. Did you have trouble thinking or remembering in the past week? (0–10), mean (SD)	3.3 (3.5)	3.6 (3.6)	+0.4	2.9 (3.6)	−0.4
13. Sensory hypersensitivity (score ≥ 5), *n* (%)	38 (39.6)	NE	NA	NE	NA
14. Were you sensitive to such things as bright lights, or loud noises, or smells in the past week? (0–10), mean (SD)	2.6 (3.6)	3.1 (3.8)	+0.5	2.5 (3.5)	+0.03
14. Sensory hypersensitivity (score ≥ 5), *n* (%)	31 (32.0)	NE	NA	NE	NA
13 & 14. Sensory hypersensitivity (score ≥ 10), *n* (%)	23 (23.7)	NE	NA	NE	NA

*Missing data for 1 patient. ^†^Laterality affects number of pain areas indicated (e.g., left hip and right hip counted as 2 regions, upper back and lower back counted as 2 regions). ^‡^One patient answered “yes” to screening pain question but responded “0” to current pain level and reported no pain areas. ^§^Percentage excluded missing responses (i.e., missing response from completed eCPQ not visits without eCPQ). For items with a number for selection, 0 indicates “not at all” or “no problem,” and 10 indicates “completely interferes” or “severe problem.” eCPQ, Electronic Chronic Pain Questions; NA, not applicable; NE, not evaluated; SD, standard deviation.

For item 10 (assessing the interference of pain on usual activities) and item 11 (assessing the impact of pain on sleep), a small increase was reported from baseline to Visit 2, but a small decrease was seen at Visit 3. For item 12 (assessing the impact of pain on mood), a small decrease was seen from baseline to both Visits 2 and 3. An increase in the scores of items 13 and 14 (assessing sensory hypersensitivity or fibromyalgia-like pain) was seen from baseline to Visit 2; at Visit 3, a decrease in the score of item 13 and a small increase in score of item 14 were reported (Table 3).

At baseline, nociceptive pain and neuropathic pain was considered for 42 (42/97, 43.3%) and 55 (55/97, 56.7%) patients, respectively; hardly any changes were found at Visit 2 (34 [34/77, 44.2%] and 43 [43/77, 55.8%], respectively). At Visit 3, 47 (47/79, 59.5%) patients had nociceptive pain and 32 (32/79, 40.5%) patients had neuropathic pain.

Fourteen patients in the Intervention Group and 9 clinicians who used the eCPQ in this study took part in the qualitative interviews exploring the feasibility of the eCPQ (interview questions see Supplementary Tables S2, S3). Although every effort was made to perform the patient interview within a 2-week window following Visit 2, due to patient scheduling, 10 patient interviews were completed outside this window. However, all 10 patients were able to recall the eCPQ during their visit. Additionally, the paper eCPQ was sent to participants prior to the telephone interviews to be available for reference throughout the interview. All patients stated that the questions were relevant to their pain experience, and frequently stated that the eCPQ was useful during their visits (12/14, 85.7%) and positively affected their physician interactions (8/14, 57.1%; e.g., “the questions… helped me probably articulate to him better what I was feeling”). Almost all patients endorsed the eCPQ questions as important to ask (12/13, 92.3%) and thought that the eCPQ would help their clinician better manage their pain (12/13, 92.3%; e.g., “you get the proper care because you can better define it”). Most patients stated they would be willing to complete the eCPQ at each visit (9/14, 64.3%) or most visits (2/14, 14.3%) (Supplementary Table S5). Most of the clinicians described a positive experience using the eCPQ (6/9, 66.7%) and described when the eCPQ would be useful in their practice (8/9, 88.9%). Although there were mixed responses regarding eCPQ training (no formal training: 4/9, 44.4%; basic training: 4/9, 44.4%), all found the eCPQ easy to use (9/9, 100%). Slightly more clinicians agreed (4/9, 44.4%) versus disagreed (3/9, 33.3%) on the extent that medical care was delivered more efficiently because of the eCPQ; however, slightly more clinicians disagreed (3/9, 33.3%) versus agreed (2/9, 22.2%) with the statement that more targeted tests and/or referrals were ordered because of the eCPQ. Almost all clinicians (6/9, 66.7%) agreed that patients who completed the eCPQ were better prepared for their visits, and that the eCPQ increased the quality of patient-physician communication (7/9, 77.8%). All clinicians agreed in some capacity that the eCPQ captures information that is pertinent to chronic pain diagnosis, treatment, and monitoring. All but 1 clinician agreed that they would like to continue using the eCPQ (Table 4).

**Table 4:** Response of the clinicians (*n* = 9) who took the qualitative interview for their perceptions of the eCPQ and its value.

	Strongly disagree	Disagree	Somewhat disagree	Neither agree nor disagree	Somewhat agree	Agree	Strongly agree
Sufficient training and support was provided on the use of the eCPQ	0	3 (33.3)	1 (11.1)	0	2 (22.2)	3 (33.3)	0
It was easy to use the information provided by the eCPQ during patient visits	0	0	0	0	2 (22.2)	3 (33.3)	4 (44.4)
Medical care was delivered more efficiently because of the eCPQ	1 (11.1)	2 (22.2)	0	2 (22.2)	1 (11.1)	3 (33.3)	0
I ordered tests and/or referrals that were more targeted because of the eCPQ	1 (11.1)	2 (22.2)	0	4 (44.4)	2 (22.2)	0	0
I changed the way I address chronic pain during patient visits because of the eCPQ	1 (11.1)	0	0	2 (22.2)	2 (22.2)	4 (44.4)	0
The eCPQ helped me to make more targeted patient care decisions	0	0	1 (11.1)	1 (11.1)	4 (44.4)	3 (33.3)	0
Patients who complete the eCPQ are better prepared for their visits	0	1 (11.1)	0	2 (22.2)	1 (11.1)	5 (55.6)	0
The eCPQ increases the quality of patient-physician communication	0	1 (11.1)	0	1 (11.1)	1 (11.1)	3 (33.3)	3 (33.3)
The eCPQ captures information that is pertinent to the diagnosis, treatment and monitoring of chronic pain	0	0	0	0	0	6 (66.7)	3 (33.3)
I would like to continue using the eCPQ as part of my clinical practice	0	1 (11.1)	0	0	3 (33.3)	5 (55.6)	0

Values are *n* (%). eCPQ, Electronic Chronic Pain Questions.

### 3.3. Patient-reported outcomes

**Figure 2:**
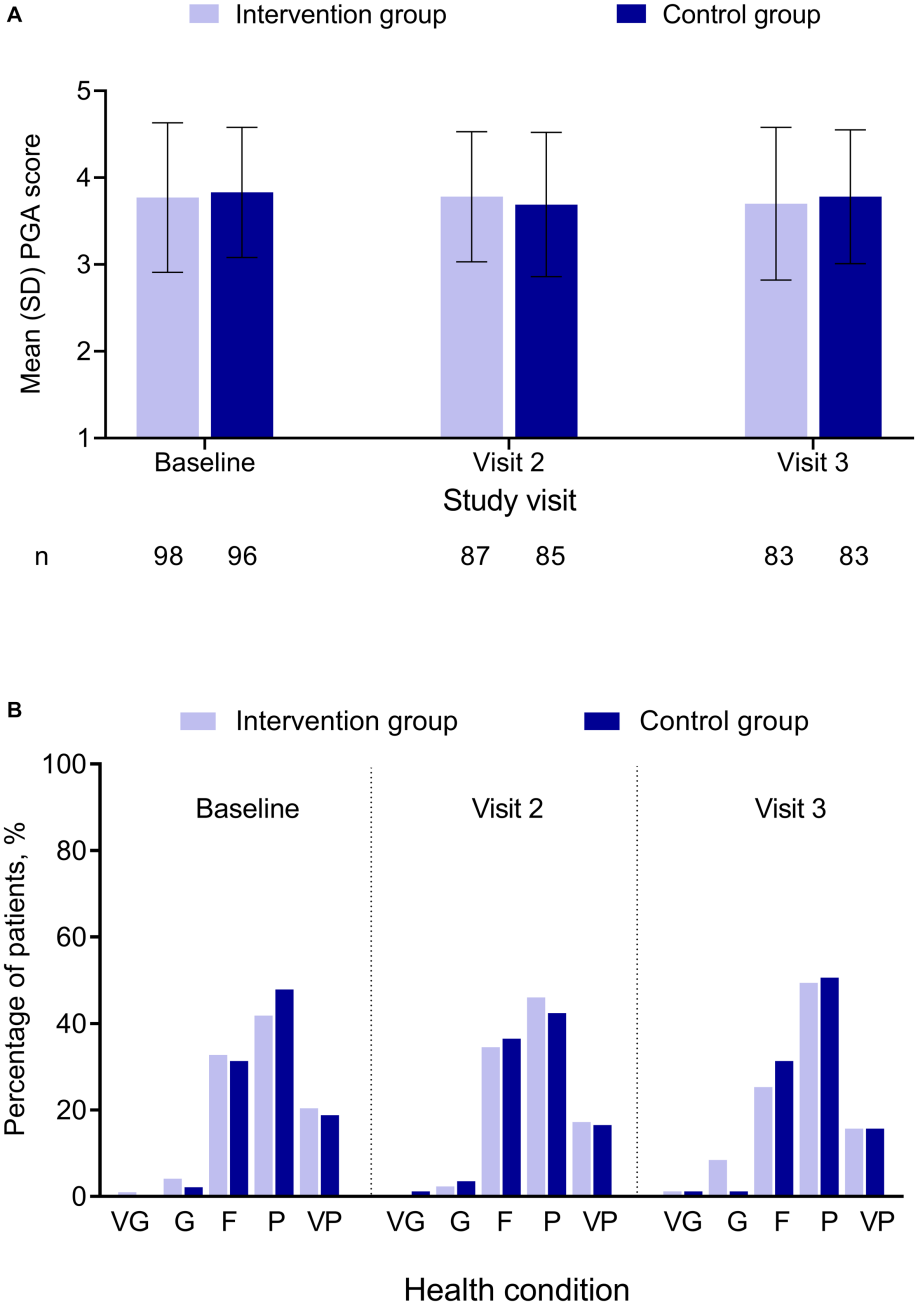
PGA by treatment group and study visit. **(A)** PGA score. **(B)** Percentage of patients by PGA category. No significant difference was found between the 2 groups. PGA score was in a 5-point Likert scale, with 1 = “very good” and 5 = “very poor.” F, fair; G, good; P, poor; PGA, Patient Global Assessment; SD, standard deviation; VG, very good; VP, very poor.

PGA score was comparable between the 2 groups for each visit, and there were minimal changes from baseline to the follow-up visits for each group (Figure 2A; Supplementary Table S6). For each visit in either group, approximately one-third of the patients reported their current state as fair and 40–50% as poor (Figure 2B; Supplementary Table S6).

**Figure 3:**
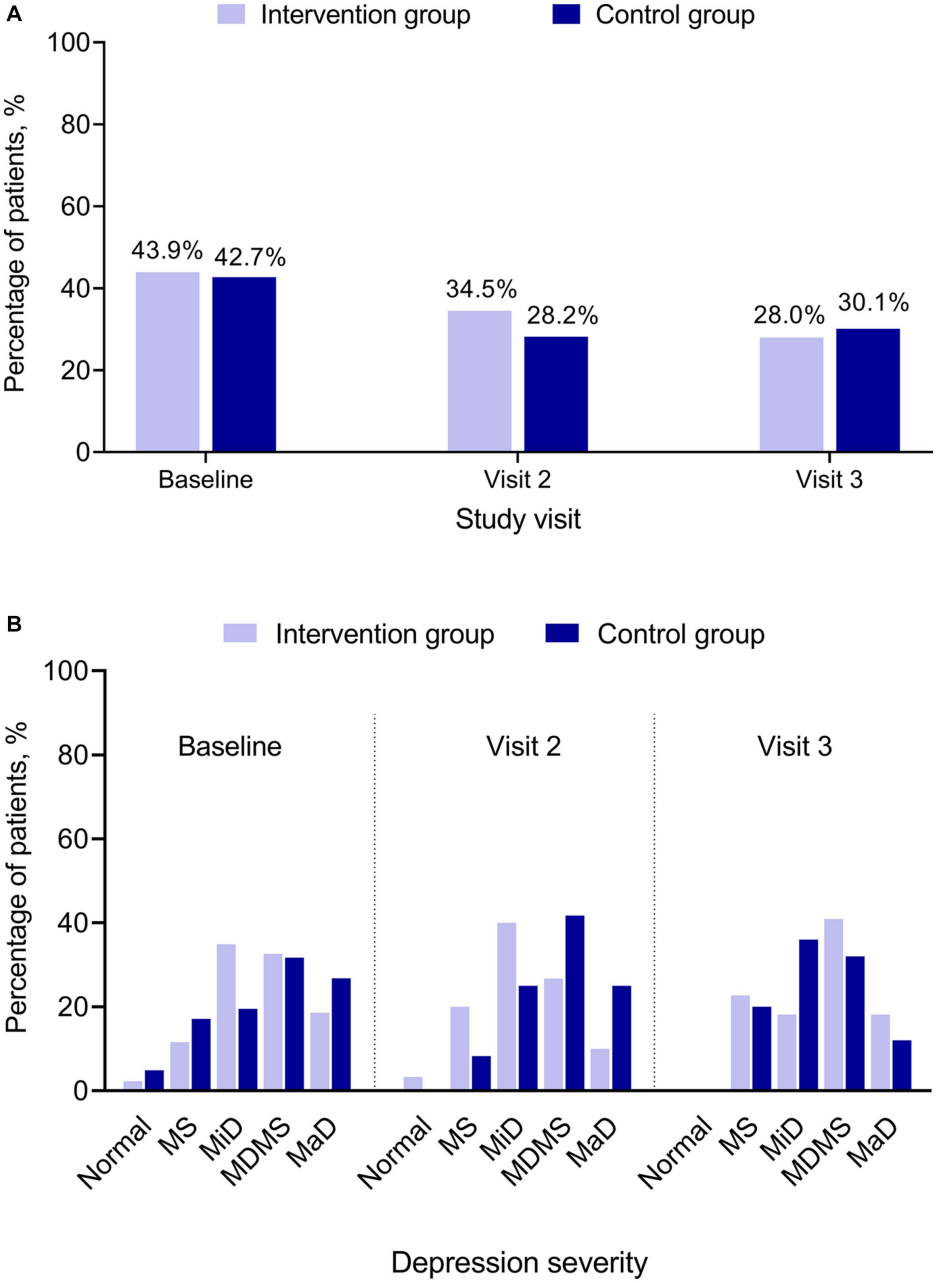
PHQ-2 and PHQ-9 by treatment group and study visit. **(A)** PHQ-2 positive. **(B)** PHQ-9 category. PHQ-2 (score range 0–6) includes the first 2 items of the PHQ-9. A score ≥3 is considered as positive indicating that the patient may be experiencing depressive symptoms. PHQ-9 (score ranges 0–27) has 9 questions. A PHQ-9 score 5–9 indicates minimal symptoms, 10–14 minor depression, 15–19 major depression moderately severe, and > 20 major depression. MaD, major depression; MDMS, major depression moderately severe; MiD, minor depression; MS, minimal symptoms; N, normal; PHQ, Patient Health Questionnaire for Depression and Anxiety (PHQ-2 and PHQ-9).

At baseline, 43.9% (43/98) of patients in the Intervention and 42.7% (41/96) in the Control Groups had positive PHQ-2 results, indicating that the patient may be experiencing depressive symptoms. The percentage of patients with positive PHQ-2 results decreased in Visits 2 and 3 for both groups (Figure 3A). At baseline, Visit 2, and Visit 3, PHQ-2 and PHQ-9 results showed no statistically significant difference between the Intervention and the Control Groups (Figure 3).

### 3.4. Healthcare resource utilization

No significant difference was found between the Intervention Group and the Control Group for any of the HCRU measures at any of the study visits (Table 5).

**Table 5:** Health services utilization.

	Intervention group *n* = 98	Control group *n* = 96
**At Visit 2 (6 months from baseline)**		
Inpatient admissions	0.1 (0.4)	0.1 (0.4)
Inpatient days	0.3 (1.2)	0.4 (1.7)
Emergency department visits	0.7 (1.1)	0.6 (1.0)
Total outpatient visits*	8.9 (8.4)	9.4 (8.2)
Primary care	2.6 (2.4)	2.4 (2.1)
Specialty care	4.5 (6.0)	5.3 (5.8)
Pain management	1.8 (3.9)	1.7 (4.2)
Total billing charges	$13,997 (21,944)	$16,176 (25,122)
**At Visit 3 (12 months from baseline)**		
Inpatient admissions	0.3 (0.7)	0.3 (0.7)
Inpatient days	1.3 (4.4)	1.0 (2.7)
Emergency department visits	1.2 (1.8)	1.2 (2.1)
Total outpatient visits*	18.6 (16.8)	18.8 (14.8)
Primary care	5.6 (4.7)	4.9 (3.7)
Specialty care	10.2 (13.0)	11.2 (11.2)
Pain management	2.8 (5.4)	2.7 (6.6)
Total billing charges	$34,726 (56,756)	$34,726 (42,402)

Values are mean (SD). No significant difference was found between the groups. *Outpatient visits were reported as total and by 3 categories, primary care (family medicine, internal medicine), pain management-related specialty care (physical therapy, occupational therapy, pain clinic, integrative [or complementary] medicine, orthopedics, sport’s medicine), and other specialty care (all other specialties not included in pain-related specialties). SD, standard deviation.

In addition, HCRU was also analyzed according to pain intensity categorized using the eCPQ data for the intervention group. At both 6 and 12 months from the baseline visit, patients with severe pain had the highest total billing charges. However, patients with moderate pain had the highest total outpatient visits, primary care visits, and pain management visits at both visits (Table 6).

**Table 6:** Health services utilization by eCPQ severity of pain category.

At 6 months from baseline visit	Intervention group		
	Mild pain *n* = 2	Moderate pain *n* = 23	Severe pain *n* = 72
Inpatient admissions	0.0 (0.0)	0.04 (0.2)	0.2 (0.5)
Inpatient days	0.0 (0.0)	0.04 (0.2)	0.4 (1.3)
Emergency department visits	0.0 (0.0)	0.6 (1.0)	0.7 (1.2)
Total outpatient visits	0.5 (0.7)	10.2 (6.8)	8.6 (8.8)
Primary care	0.0 (0.0)	3.7 (2.8)	2.3 (2.3)
Specialty care	0.5 (0.7)	4.5 (3.1)	4.7 (6.8)
Pain management	0.0 (0.0)	2.1 (4.8)	1.7 (3.5)
Total billing charges	$1,903 (2,551)	$10,895 (11,678)	$15,388 (24,618)

At 12 months from baseline visit	Mild pain *n* = 2	Moderate pain *n* = 23	Severe pain *n* = 72
Inpatient admissions	0.0 (0.0)	0.1 (0.3)	0.4 (0.8)
Inpatient days	0.0 (0.0)	0.2 (0.5)	1.7 (5.1)
Emergency department visits	0.5 (0.7)	1.2 (1.9)	1.2 (1.8)
Total outpatient visits	3.0 (4.2)	19.7 (11.4)	18.2 (18.1)
Primary care	0.0 (0.0)	6.9 (5.7)	5.3 (4.3)
Specialty care	3.0 (4.2)	9.8 (6.8)	10.4 (14.6)
Pain management	0.0 (0.0)	3.0 (6.0)	2.5 (4.7)
Total billing charges	$3,008 (2,733)	$23,675 (24,515)	$38,564 (61,854)

Values are mean (standard deviation). eCPQ, Electronic Chronic Pain Questions.

## 4. Discussion

Using the eCPQ as part of the primary care for patients with chronic pain who had visited the clinics every 6 months showed limited overall impact of pain on normal daily activities as measured by the PGA. Management of chronic pain is often a long-term process with multimodal and multidisciplinary interventions as it is difficult to change treatment outcomes once the patient has long-standing chronic pain. For a subgroup of patients with chronic pain, an intractable pain syndrome or disease has been reported (26). Pain management plans on average only achieve 30–40% pain reduction in fewer than half of treated patients (27). These might have contributed to our showing minimal or no improvement in PGA scores. Further, the eCPQ assessments indicated a much higher prevalence of neuropathic pain (57% at baseline and 41% at the 1-year follow-up visit) in the study population compared with what has been reported previously (7–10%) in the general population (28–30). This might partially explain the reason why no great improvement was observed on clinical outcomes, as the efficacy of most available treatments for neuropathic pain is moderate (30% improvement) (31).

Qualitative interviews with patients and clinicians who were in the Intervention Group and used the eCPQ supported the content of the eCPQ. Overall, the eCPQ was a well-accepted and potentially useful tool from the patient and the clinician’s perspective, which is similar to the qualitative feedback received previously (24). Most patients reported that the eCPQ was relevant, useful, and important. They also reported that the eCPQ affected treatment satisfaction and it could help better manage their pain, had a positive effect on their clinician interactions, and could improve the quality of care. Therefore, most patients were willing to complete the eCPQ at their clinic visits. Positive feedback was received from the clinicians as well. Most clinicians agreed that the eCPQ captures information that is pertinent to chronic pain diagnosis, treatment, and monitoring. They acknowledged that there were instances when the eCPQ would be useful and patients who completed the eCPQ were better prepared for their visits. Hence, they would like to continue using the eCPQ as part of their clinical practice. The interpersonal relationship of the patient and the healthcare provider has been acknowledged as critical for treatment success and patient satisfaction with care (32). In particular, primary care providers’ behaviors, for example, spending time to listen to all concerns of the patients, having active communication and being responsive, and offering comprehensive pain management, were directly related to high patient satisfaction (33). Although patient satisfaction was not measured in the current analysis, the findings of the interviews suggested that using the eCPQ would have a potential benefit to improving patient satisfaction of care.

It has been observed that in the follow-up visits, the percentage of patients with positive PHQ-2 showed a decrease from baseline. Since there are only 2 items in PHQ-2 and the patients might have guessed the relationship between their response and the positive results after the baseline visit, it was possible that the patients deliberately chose their response in Visits 2 and 3 to avoid a positive result indicating depression, since a positive result would lead to unwanted additional follow-up for a possible diagnosis of depression. Further, there is no clear trend in the PHQ-9 categories between the Intervention Group and the Control Group across the study period. No definite conclusion could be made on the effect of eCPQ on PHQ-9 due to the small sample size.

For patients with osteoarthritis, increased pain severity is associated with increased HCRU, such as increased number of imaging tests used to monitor osteoarthritis progression, more healthcare provider visits, higher hospitalization rates, and more prior and planned surgeries (34). In the current study, analysis of HCRU at 6 months and 12 months from baseline showed that, compared with patients categorized as having severe pain based on the eCPQ in the intervention group, those having moderate pain had numerically higher total outpatient visits, outpatient visits–primary care, and outpatient visits–pain management. It could be speculated that the usage of eCPQ might have some influence on patient behavior in seeking medical care for chronic pain, and it might also have influenced the clinicians’ decision making in managing chronic pain. It should also be noted that since the sample size was calculated based on assumptions made for PGA score, the study could be underpowered for comparisons of HCRU outcomes.

This study was carried out in an urban clinic. The participating patients were predominantly (>85%) Black/African American, which mirrored the demographic of the population served by these urban clinics. Baseline demographic characteristics, findings in PROs, and assessments of HCRU were similar between the two groups; no other analysis was performed. There are limitations of the current study. Patients who completed the eCPQ reported many body areas affected by chronic pain with back pain and pain in the lower extremity as the most common and no details to differentiate joint pain from soft tissue pain. Therefore, it might not be possible to extrapolate the results to patients with other types of chronic pain. Further, patients included in the current study were likely to have long-standing pain conditions (at least lasting for more than 3 months), and it is not clear whether implementing the eCPQ upon diagnosis of chronic pain versus an entrenched patient population would make a difference in clinical outcomes. Also, this study did not capture data to describe clinical decisions for treatment changes based on the provider having access to eCPQ. For instance, no data were collected that could show whether patients with neuropathic pain as identified by the eCPQ had received appropriate medication choices and doses, or whether there were changes in treatment regime based on the type of pain identified by the eCPQ. Considering that any changes in clinical decision making and its potential causal factors would be of significant interest to clinicians, obtaining such data should be evaluated and included, if at all feasible, in the design of future studies. All of these might have limited the interpretation of the results. In this study, patients completed a paper version of the eCPQ and the research team entered the results into the electronic medical records; such a process may limit the generalizability and utility of the eCPQ in a busy clinical settings without a research team. The qualitative interview was only conducted on a proportion of the participants in the eCPQ group. Nevertheless, even though the sample size was small, results of the qualitative interviews performed in patients with chronic pain and physicians who used the eCPQ indicated that it was beneficial using the eCPQ in clinical practice for chronic pain management, as these patients who completed the eCPQ were better prepared for their visits and the quality of patient-physician communication was increased compared with those who received regular care only.

In summary, adding eCPQ to the regular care for patients with chronic pain attending a primary care practice did not significantly impact PROs. However, qualitative interviews suggested that the eCPQ was a well-accepted and potentially useful tool from the patient and physician perspective. More detailed investigation is warranted to determine the usefulness of the eCPQ.

## Data availability statement

The original contributions presented in the study are included in the article/Supplementary material. Upon request, and subject to review, Pfizer will provide the data that support the findings of this study. Subject to certain criteria, conditions and exceptions, Pfizer may also provide access to the related individual de-identified participant data. See https://www.pfizer.com/science/clinical-trials/trial-data-and-results for more information.

## Ethics statement

The studies involving human participants were reviewed and approved by the Institutional Review Board at the Henry Ford Health (HFH). The study was conducted in compliance with the Declaration of Helsinki. The patients/participants provided their written informed consent to participate in this study.

## Author contributions

LL, VS, LA, JC, BD, SE, RH-D, and PP contributed to research idea, study design, data analysis, data interpretation, manuscript development, manuscript review, and approval for manuscript submission. LL, VS, and SE contributed to data acquisition. LL contributed to statistical analysis. All authors contributed to the article and approved the submitted version.

## Funding

This study was sponsored by Pfizer. The qualitative substudy in patients and physicians was conducted by Evidera and was sponsored by Pfizer. Employees of the funding source, Pfizer, who contributed to the design of the study, the collection, management, analysis, and interpretation of the data, and reviewed the manuscript, are named authors of this manuscript.

## Conflict of interest

LL, VS, and SE are employees of the Henry Ford Health, which received funding from Pfizer for this study. LA, JC, BD, RH-D, and PP are employees of Pfizer with stock and/or stock options.

## Publisher’s note

All claims expressed in this article are solely those of the authors and do not necessarily represent those of their affiliated organizations, or those of the publisher, the editors and the reviewers. Any product that may be evaluated in this article, or claim that may be made by its manufacturer, is not guaranteed or endorsed by the publisher.

## Footnotes

^1^©Pfizer Inc. All rights reserved. The questionnaire is available in different languages, which can be obtained at https://www.pfizerpcoa.com/chronic-pain-questions-cpq-screener. There is no charge for academic research/non-profit organizations and individual clinical practice; for commercial research/industry sponsored trials and studies, there is a charge to pay. More details can be found at the website or by contacting Pfizer directly.
