# Supplementary material for: Impact of Electronic Chronic Pain Questions on patient-reported outcomes and healthcare utilization, and attitudes toward eCPQ use among patients and physicians: prospective pragmatic study in a US general practice setting
Source: Front Med (Lausanne). 2023 Jun 22;10:933975. doi: 10.3389/fmed.2023.933975 (PMC10323749; doi:10.3389/fmed.2023.933975)
Supplement: Supplementary file 1 [file Data_Sheet_1.pdf]

Supplementary Materials

Impact of Electronic Chronic Pain Questions (eCPQ) on Patient-Reported Outcomes and Healthcare Utilization, and Attitudes Towards eCPQ Use Among Patients and Physicians: Prospective Pragmatic Study in a US General Practice Setting

Lois Lamerato et al.

Contents

Table S1. ICD-10-CM codes used to identify eligible patients 2
Table S2. Questions asked during the patient qualitative interview* 3
Table S3. Questions asked during the clinician qualitative interview 5
Table S4. Baseline pain areas according to eCPQ 7
Table S5. Findings of the qualitative patient interview 8
Table S6. Changes in PGA score 12
Figure S1. Schematic of the eCPQ 13
Figure S2. Patient population 14

Table S1. ICD-10-CM codes used to identify eligible patients

ICD-10-CM Code	Diagnosis
G89.2	Chronic pain, not elsewhere classified
G89.29	Other chronic pain
G89.4	Chronic pain syndrome

ICD-10-CM, International Classification of Diseases, 10th edition, Clinical Modification.

Table S2. Questions asked during the patient qualitative interview*

**About the eCPQ[1]**	
1	What did you think about the questionnaire overall?
2	How relevant were these questions to your experiences with pain?
3	What did you think about the amount of time it took to complete the CPQ in the exam room before your clinic visit?
4	What did you think about completing the questionnaire before your clinic visit?
**eCPQ intervention**	
1	How did completing the CPQ affect your interactions with your physician?
2	Did you find the CPQ useful in your visit with your physician? How so?
3	What benefits did you experience because you completed the CPQ?
4	What limitations or disadvantages did you experience because you completed the CPQ?
5	Did you feel that the CPQ improved the quality of the care you received? If so, how?
6	How did the CPQ affect how satisfied you felt with the service/treatment you received from your physician?
7	Are these questions important to ask at scheduled appointments? Why?
8	Do you think the CPQ will help you and your clinician better manage your pain? Why?
9	Would you be willing to complete the CPQ each time you came to see your physician? Why or why not?
10	How could we further improve your interactions with clinicians related to your pain?
**eCPQ score change from baseline to Visit 2**	
1	Your eCPQ score at your most recent clinic visit was [X – based on clinician report]. In contrast, your eCPQ score at your baseline visit was [X – based on clinician report]. Can you tell me about what differences you experienced in your pain at each of these visits?
2	Were you able to accurately capture or summarize your pain experience at each time with the eCPQ? How so?
**Conclusion**	
	Are there any other thoughts related to completing the CPQ that we did not discuss today? If so, tell me more about that.

* Eligible patients were required to be able to speak, read, and write English sufficiently well to complete and self-administer study questionnaires. The patient qualitative interview was conducted

[1]. ©Pfizer Inc. All rights reserved. The questionnaire is available in different languages, which can be obtained at https://www.pfizerpcoa.com/chronic-pain-questions-cpq-screener. There is no charge for academic research/non-profit organizations and individual clinical practice; for commercial research/industry sponsored trials and studies, there is a charge to pay. More details can be found at the website or by contacting Pfizer directly.

via telephone. Interview materials (i.e., the paper eCPQ and sociodemographic questionnaire) were sent to patient participants prior to the interview. Interviews were conducted by an experienced and trained interviewer. All interviews were recorded after the participant verbally gave permission to be recorded. Interviews lasted no more than 30 minutes, and participants completed a brief sociodemographic questionnaire upon completion of the interview. The brief sociodemographic questionnaire gathered basic information on age, gender, race/ethnicity, employment status, education level, date of pain symptom onset, and comorbidities.

The qualitative data were analyzed with ATLAS.ti software version 8.4.20.0 to facilitate the systematic coding and analysis of the data. Qualitative data from the interview discussions were reviewed by the interviewer, and key themes that described important concepts raised by participants were identified. An a priori coding dictionary was developed based on themes and concepts that emerged during the interviews.

eCPQ, electronic Chronic Pain Questions.

Table S3. Questions asked during the clinician qualitative interview

**Professional development**	
1	What is your degree?
2	What is your specialty?
3	About how long ago did you recruit a patient to the eCPQ study?<br>[Only clinicians who have used the eCPQ within the last three months will continue with the interview].
4	How long have you been working with chronic pain patients?
5	Roughly how many patients with chronic pain do you see per month on average?
6	Can you give a general overview of your experience using the eCPQ as part of your clinical practice? [Probe if needed: (1) What has worked about the eCPQ study? (2) What challenges have you encountered implementing the eCPQ study?]
**eCPQ – post-study provider survey**<br>Clinicians need to make one selection from the choices below for each topic: (1) strongly disagree, (2) disagree, (3) somewhat disagree, (4) neither agree nor disagree, (5) somewhat agree, (6) agree, and (7) strongly agree.	
1	Sufficient training and support was provided on the use of the eCPQ<br><u>Interview question</u>: Why did you select [response] for this question? [Probe if needed: (1) What kinds of training did you receive? (2) What did you like/dislike about the training?]
2	It was easy to use the information provided by the eCPQ during patient visits<br><u>Interview question:</u> Why did you select [response] for this question? [Probe if needed: Can you describe how you used information provided by the eCPQ during patient visits?]
3	Medical care was delivered more efficiently because of the eCPQ<br><u>Interview question:</u> Why did you select [response] for this question? [Probe if needed: Can you describe how the eCPQ did/did not make medical care delivery more efficient?]
4	I ordered tests and/or referrals that were more targeted because of the eCPQ<br><u>Interview question:</u> Why did you select [response] for this question? [Probe if needed: How did you use the eCPQ to determine tests and/or referrals?]
5	I changed the way I address chronic pain during patient visits because of the eCPQ<br><u>Interview question:</u> Why did you select [response] for this question? [Probe if needed: How did the eCPQ change the way you addressed chronic pain during patient visits, if at all?]
6	The eCPQ helped me to make more targeted patient care decisions

	Interview question: Why did you select [response] for this question? [Probe if needed: Can you describe how the eCPQ did/did not help you make more targeted patient care decisions?]
7	Patients who complete the eCPQ are better prepared for their visits<br>Interview question: Why did you select [response] for this question? [Probe if needed: Do you agree with this statement? Why or why not?]
8	The eCPQ increases the quality of patient-physician communication<br>Interview question: Why did you select [response] for this question? [Probe if needed: Do you agree with this statement? Why or why not?]
9	The eCPQ captures information that is pertinent to the diagnosis, treatment and monitoring of chronic pain<br>Interview question: Why did you select [response] for this question? [Probe if needed: Do you agree with this statement? Why or why not?]
10	I would like to continue using the eCPQ as part of my clinical practice<br>Interview question: Why did you select [response] for this question? [Probe if needed: Do you agree with this statement? Why or why not?]
**Future contact**	
1	If we have any brief follow-up questions, is it okay to contact you by e-mail or telephone?

eCPQ, electronic Chronic Pain Questions.

Table S4. Baseline pain areas according to eCPQ

Pain area	Number of responses	Frequency (%)*
Back	66	69.5
Lower extremity	66	69.5
Upper extremity	18	18.9
Wrist/hand	22	23.2
Ankle/foot	28	29.5
Shoulder	31	32.6
Hip	29	30.5
Neck	12	12.6
Abdomen	10	10.5
Other	16	16.8

* Of the 98 patients in the Intervention Group, eCPQ data were available for 97 (1 patient missing eCPQ data), 95 of whom indicated pain areas. Laterality was not considered (e.g., right foot and left foot reported as foot) when the frequency (%) of responses to specific body areas affected by pain was calculated.

eCPQ, electronic Chronic Pain Questions.

Table S5. Findings of the qualitative patient interview

	**Patient, *n* (%)**
**Overall perception of the eCPQ (*n* = 14)**	
General feedback of the eCPQ	
Positive	7 (50.0)
Neutral	7 (50.0)
The eCPQ was helpful in understanding what patients are going through with chronic pain	3 (21.4)
The eCPQ had overall good, thorough questions	3 (21.4)
The eCPQ questions were relevant to the pain experience	14 (100)
The amount of time it took to complete the eCPQ	
Positive	3 (21.4)
Neutral	10 (71.4)
Time spent on completing the eCPQ was less than 30 min, and did not want to spend longer than that	1 (7.1)
No direct comment	1 (7.1)
About completing the eCPQ before the clinic visit (*n* = 12)	
Negative (i.e., it was difficult to focus on the eCPQ questions while in pain, which was the reason for seeking medical help at HFH). It was preferred to address the pain and not complete the eCPQ	1 (8.3)
**eCPQ affected physician interactions (*n* = 14)**	
Positive	8 (57.1)
The eCPQ could help with pain understanding for the patient and the clinician	5 (35.7)
Spending more time with the physician	1 (7.1)
The physician suggested exercise and therapy to manage the pain after reviewing the eCPQ	1 (7.1)
The eCPQ helped the participant remember what to ask the physician	1 (7.1)
Neutral	2 (14.3)
Did not affect the interaction	4 (28.6)
**eCPQ useful in visit with physician (*n* = 14)**	
Positive	12 (85.7)
The eCPQ helped them articulate or explain their pain to their physician, or ask the physician things about their pain that they would normally not have asked the physician	7 (50.0)
Neutral (did not have the eCPQ when talking to the physician)	1 (7.1)
Was not useful	1 (7.1)
**eCPQ benefits versus limitation/disadvantages (*n* = 13)**	
Positive	9 (69.2)

Being able to explain the pain to the physician	3 (23.1)
Increased understanding or knowledge about the pain	2 (15.4)
Better identification of the pain	2 (15.4)
Better care or medication	2 (15.4)
Did not experience any benefits from the eCPQ	4 (30.8)
No disadvantages from completing the eCPQ	9 (69.2)
Limitations of completing the eCPQ (i.e., having to remember the pain experience and completing the eCPQ when being late for the appointment)	2 (15.4)
**eCPQ improved quality of care (*n* = 12)**	
Positive	6 (50.0)
Improved the education or understanding about pain	3 (25.0)
Improved collaboration with the physician	1 (8.3)
Prompted recalling pain at the time of the visit	1 (8.3)
Helped prescribe better pain medication	1 (8.3)
Quality of care remained the same	2 (16.7)
Not sure	2 (16.7)
Did not improve quality of care	2 (16.7)
**eCPQ affecting service/treatment satisfaction (*n* = 13)**	
Positive	8 (61.5)
Helping the doctor understand pain better	2 (15.4)
Targeting pain areas	1 (7.7)
Felt more listened to by completing the eCPQ	1 (7.7)
Felt the doctor and nurse were more concerned with the healthcare	1 (7.7)
New questions never asked before	1 (7.7)
No reasons given	2 (15.4)
Did not affect treatment satisfaction	5 (38.5)
**eCPQ important to ask (*n* = 13)**	
Positive	12 (92.3)
Helped the physician understand the pain	5 (38.5)
Helped define the pain	3 (23.1)
Covered topics that may not be asked normally	1 (7.7)
Helped physician and patient collaboration	1 (7.7)
Helped diagnose new conditions	1 (7.7)
Helped the doctor become familiar with new patients	1 (7.7)

The eCPQ would only be important to ask depending on the level of pain; the individual would not want to be asked these questions when having more pain	1 (7.7)
**eCPQ helping better manage pain (*n* = 13)**	
Positive	12 (92.3)
Receiving better treatment specific to the pain	5 (38.5)
Better communication with physicians	3 (23.1)
Better pain evaluation, definition, or identification	2 (15.4)
Better identifying the effects of pain	1 (7.7)
No reason given	1 (7.7)
The eCPQ could not help better manage their pain. However, the eCPQ was a useful tool	1 (7.7)
**Willingness to complete eCPQ and improving clinician interactions (*n* = 14)**	
Positive	
Willing to complete the eCPQ at every appointment with the physician	9 (64.3)
Willing to complete the eCPQ most of the time	2 (14.3)
The eCPQ could help with understanding their pain	2 (14.3)
The doctor could suggest actions based on the eCPQ	2 (14.3)
The eCPQ data could help others in pain	1 (7.1)
The answers could be compared to other visits	1 (7.1)
Seeing the doctor infrequently	1 (7.1)
The pain may have changed	1 (7.1)
Neutral	
The level of pain or exhaustion would affect the willingness to complete the eCPQ	1 (7.1)
Depending on the individual as some people may just want to have an appointment with the doctor to have their problems treated and then leave the clinic	1 (7.1)
Not willing to complete the eCPQ at each appointment as the eCPQ might not be necessary at every visit and it could be completed annually or semi-annually	3 (21.4)
**What could be done to further improve the interactions with clinicians related to the pain (*n* = 12)**	
Interactions could be improved	5 (41.7)
Treating as individuals versus a group	1 (8.3)
Through personalized medicine	1 (8.3)
Doctors' learning from new pains	1 (8.3)
Doctors asking questions based on the eCPQ to further understand pain	1 (8.3)
Asking questions about pain medications	1 (8.3)
Nothing was needed to improve the clinician interactions	7 (58.3)

**eCPQ score change and accuracy (*n* = 14)**	
eCPQ scores increased (worsened) from baseline to Visit 2	8 (57.1)
eCPQ scores decreased (improved) from baseline to Visit 2	5 (35.7)
eCPQ score remained the same	1 (7.1)
Differences in pain related to:	
Differences in weather	4 (28.6)
Activity or body positions	2 (14.3)
Pain being treated	2 (14.3)
Pain being different daily	2 (14.3)
Not enough rest or sleep	1 (7.1)
Medication not working	1 (7.1)
Pain intensified	1 (7.1)
Different pain or different pain area	1 (7.1)
Not sure what pain differences contributed to increased eCPQ score	1 (7.1)
Ability to accurately capture or summarize pain experience each time when completing the eCPQ	
Positive	9 (64.3)
The eCPQ specified pain	3 (21.4)
The eCPQ covered common pain experiences	2 (14.3)
The eCPQ was easy for the user to recall the pain	2 (14.3)
The eCPQ was easy to use	1 (7.1)
Not be able to as the questions being more general than specific and pain differences affecting answers	2 (14.3)
The eCPQ could sometimes accurately capture pain experience due to the pain differing	1 (7.1)
Not sure if the eCPQ could accurately capture the pain experience differing	1 (7.1)
Did not seem to understand the probe	1 (7.1)

eCPQ, electronic Chronic Pain Questions; HFH, Henry Ford Health.

Table S6. Changes in PGA score

	Cases		Controls	
	**n**	**Mean (SD)**	**n**	**Mean (SD)**
**PGA**				
Visit 1	98	3.77 (0.86)	96	3.83 (0.75)
Visit 2	87	3.78 (0.75)	85	3.69 (0.83)
Visit 3	83	3.70 (0.88)	83	3.78 (0.77)

No significant difference was found between the 2 groups.

PGA score was in a 5-point Likert scale, with 1 = “very good” and 5 = “very poor”.

PGA, Patient Global Assessment; SD, standard deviation.

Figure S1. Schematic of the eCPQ

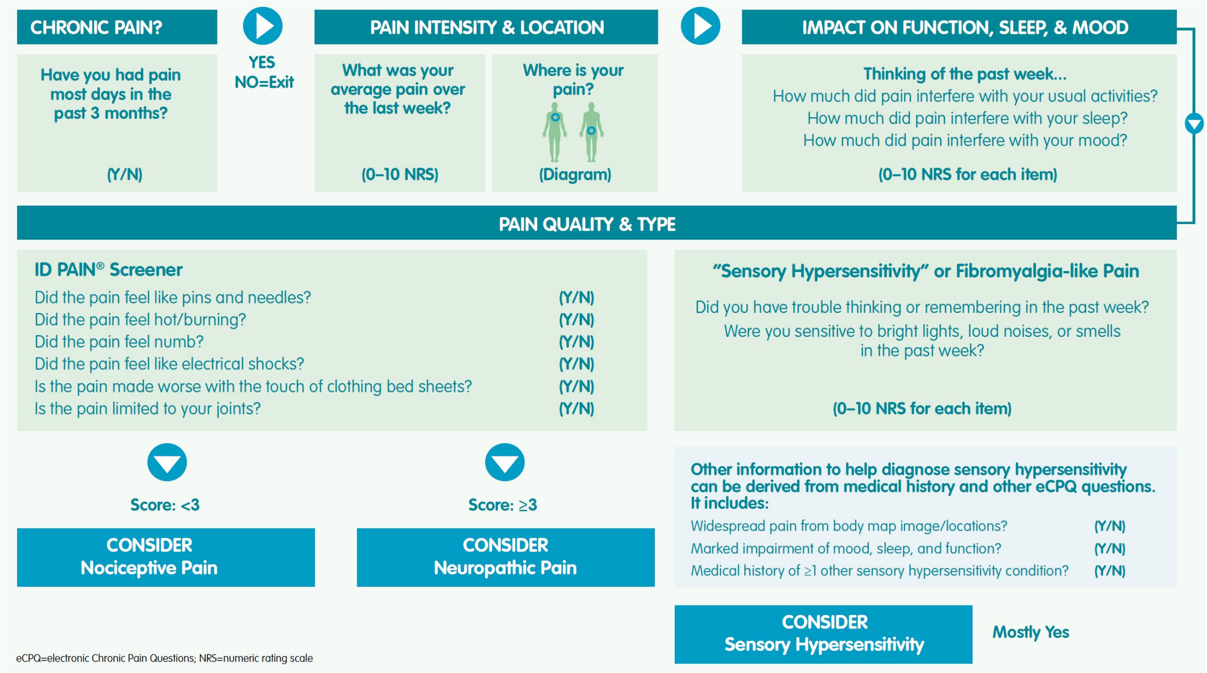

eCPQ, electronic Chronic Pain Questions; NRS, numeric rating scale.

Figure S2. Patient population

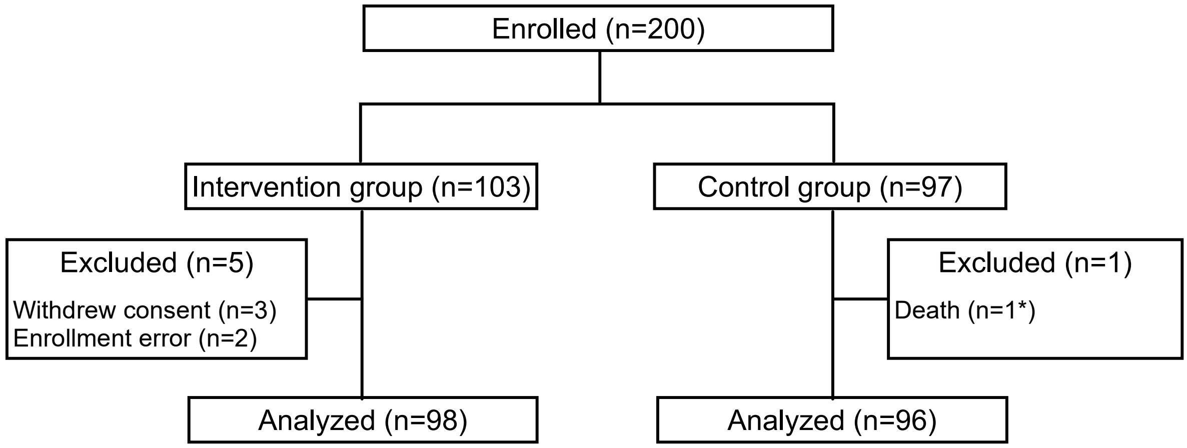

* This patient had severe illness (Stage 4 sarcoidosis, multiple comorbidities) and died after being hospitalized for 96 days. The death occurred prior to Visit 3.
